# Supplementary material for: Genetic Bias, Diversity Indices, Physiochemical Properties and CDR3 Motifs Divide Auto-Reactive from Allo-Reactive T-Cell Repertoires
Source: Int J Mol Sci. 2021 Feb 5;22(4):1625. doi: 10.3390/ijms22041625 (PMC7915266; doi:10.3390/ijms22041625)
Supplement: Supplementary file 1 [file ijms-22-01625-s001.pdf]

Supplementary Table 1

HLA-A2+ IMNDMPIYM clones

Donor	HLA-A0201	TRAV	CDR3	TRAJ	TRBV	CDR3	TRBJ
Donor 1	Positive	TRAV9-2	CALSDSWGKFQF	TRAJ24	TRBV2	CARRLAGEYNEQFF	TRBJ2-1
Donor 1	Positive	TRAV12-2	CAVYDGQKLLF	TRAJ16	TRBV5-4	CASSSDNTAGELFF	TRBJ2-2
Donor 1	Positive	TRAV12-2	CAVYDGQKLLF	TRAJ16	TRBV5-4	CASSSDNTAGELFF	TRBJ2-2
Donor 1	Positive	TRAV12-2	CAVYDGQKLLF	TRAJ16	TRBV5-4	CASSSDNTAGELFF	TRBJ2-2
Donor 1	Positive	TRAV12-2	CAVYDGQKLLF	TRAJ16	TRBV5-4	CASSSDNTAGELFF	TRBJ2-2
Donor 1	Positive	TRAV12-2	CAVYDGQKLLF	TRAJ16	TRBV5-4	CASSSDNTAGELFF	TRBJ2-2
Donor 1	Positive	TRAV12-2	CAVYDGQKLLF	TRAJ16	TRBV5-4	CASSSDNTAGELFF	TRBJ2-2
Donor 1	Positive	TRAV12-2	CAVYDGQKLLF	TRAJ16	TRBV5-4	CASSSDNTAGELFF	TRBJ2-2
Donor 1	Positive	TRAV12-2	CAVYDGQKLLF	TRAJ16	TRBV5-4	CASSSDNTAGELFF	TRBJ2-2
Donor 1	Positive	TRAV12-2	CAVYDGQKLLF	TRAJ16	TRBV5-4	CASSSDNTAGELFF	TRBJ2-2
Donor 1	Positive	TRAV12-2	CAVYDGQKLLF	TRAJ16	TRBV5-4	CASSSDNTAGELFF	TRBJ2-2
Donor 1	Positive	TRAV12-2	CAVYDGQKLLF	TRAJ16	TRBV5-4	CASSSDNTAGELFF	TRBJ2-2
Donor 1	Positive	TRAV12-2	CAVYDGQKLLF	TRAJ16	TRBV5-4	CASSSDNTAGELFF	TRBJ2-2
Donor 1	Positive	TRAV12-2	CAVYDGQKLLF	TRAJ16	TRBV5-4	CASSSDNTAGELFF	TRBJ2-2
Donor 1	Positive	TRAV12-2	CAVYDGQKLLF	TRAJ16	TRBV5-4	CASSSDNTAGELFF	TRBJ2-2
Donor 1	Positive	TRAV12-2	CAVYDGQKLLF	TRAJ16	TRBV5-4	CASSSDNTAGELFF	TRBJ2-2
Donor 1	Positive	TRAV12-2	CAVYDGQKLLF	TRAJ16	TRBV5-4	CASSSDNTAGELFF	TRBJ2-2
Donor 1	Positive	TRAV12-2	CAVYDGQKLLF	TRAJ16	TRBV5-4	CASSSDNTAGELFF	TRBJ2-2
Donor 1	Positive	TRAV12-2	CAVYDGQKLLF	TRAJ16	TRBV5-4	CASSSDNTAGELFF	TRBJ2-2
Donor 1	Positive	TRAV12-2	CAVYDGQKLLF	TRAJ16	TRBV5-4	CASSSDNTAGELFF	TRBJ2-2
Donor 1	Positive	TRAV12-2	CAVYDGQKLLF	TRAJ16	TRBV5-4	CASSSDNTAGELFF	TRBJ2-2
Donor 1	Positive	TRAV12-2	CAVYDGQKLLF	TRAJ16	TRBV5-4	CASSSDNTAGELFF	TRBJ2-2
Donor 1	Positive	TRAV12-2	CAVYDGQKLLF	TRAJ16	TRBV5-4	CASSSDNTAGELFF	TRBJ2-2
Donor 1	Positive	TRAV12-2	CAVYDGQKLLF	TRAJ16	TRBV5-4	CASSSDNTAGELFF	TRBJ2-2
Donor 1	Positive	TRAV12-2	CAVYDGQKLLF	TRAJ16	TRBV5-4	CASSSDNTAGELFF	TRBJ2-2
Donor 1	Positive	TRAV12-2	CAVYDGQKLLF	TRAJ16	TRBV5-4	CASSSDNTAGELFF	TRBJ2-2
Donor 1	Positive	TRAV12-2	CAVYDGQKLLF	TRAJ16	TRBV5-4	CASSSDNTAGELFF	TRBJ2-2
Donor 1	Positive	TRAV12-2	CAVYDGQKLLF	TRAJ16	TRBV5-4	CASSSDNTAGELFF	TRBJ2-2
Donor 1	Positive	TRAV12-2	CAVYDGQKLLF	TRAJ16	TRBV5-4	CASSSDNTAGELFF	TRBJ2-2
Donor 1	Positive	TRAV12-2	CAVYDGQKLLF	TRAJ16	TRBV5-4	CASSSDNTAGELFF	TRBJ2-2
Donor 1	Positive	TRAV12-2	CAVYDGQKLLF	TRAJ16	TRBV5-4	CASSSDNTAGELFF	TRBJ2-2
Donor 1	Positive	TRAV12-2	CAVYDGQKLLF	TRAJ16	TRBV5-4	CASSSDNTAGELFF	TRBJ2-2
Donor 1	Positive	TRAV19	CALSEASRDDKIIF	TRAJ30	TRBV7-9	CASSPDREWETQYF	TRBJ2-5
Donor 2	Positive	TRAV19	CALISNQAGTALIF	TRAJ15	TRBV11-2	CASSLEGLAGAGWYQETQYF	TRBJ2-5
Donor 2	Positive	TRAV5	CAESPWMDSSYKLIF	TRAJ15	TRBV11-2	CASSPVGGDTEAFF	TRBJ1-1
Donor 2	Positive	TRAV12-3	CAMTGYQKVTF	TRAJ13	TRBV11-3	CASSPPNRLDEQFF	TRBJ2-1
Donor 2	Positive	TRAV19	CALIEMYSGGGADGLTF	TRAJ45	TRBV12-3	CASSPRTDRSGANVLTF	TRBJ2-6
Donor 2	Positive	TRAV19	CALSGLMDSSYKLIF	TRAJ12	TRBV19	CASSIGVQLYGYTF	TRBJ1-2
Donor 2	Positive	TRAV19	CALSEALDNQGGKLIF	TRAJ23	TRBV19	CASSILGSYNEQFF	TRBJ2-1
Donor 2	Positive	TRAV27	CAGADFTGTASKLTF	TRAJ44	TRBV19	CASTGFRTSGISYNEQFF	TRBJ2-1
Donor 2	Positive	TRAV35	CAGQTYTGTASKLTF	TRAJ44	TRBV19	CASSSTGDTYGYTF	TRBJ1-2
Donor 2	Positive	TRAV8-1	CAVNAWTGTASKLTF	TRAJ44	TRBV19	CASSSNRGPEQFF	TRBJ2-1
Donor 2	Positive	TRAV10	CVTTDSWGKLQF	TRAJ24	TRBV2	CASSGAQGYEQYF	TRBJ2-7
Donor 2	Positive	TRAV17	CATAGGLPMNYGGATNKLIF	TRAJ32	TRBV2	CASLWTGETYEQYF	TRBJ2-7
Donor 2	Positive	TRAV25	CAGVDSWGKFQF	TRAJ24	TRBV2	CASRILAGGIYEQYF	TRBJ2-7
Donor 2	Positive	TRAV38-2	CAYRSFRDDKIIF	TRAJ30	TRBV2	CASSDPRTGFHEQYF	TRBJ2-7
Donor 2	Positive	TRAV8-2	CVVSDSWGKLQF	TRAJ24	TRBV2	CASSDSRGSNSPLHF	TRBJ1-6
Donor 2	Positive	TRAV12-2	CAVKEWGKLVF	TRAJ47	TRBV20-1	CSARDQSSNQPQHF	TRBJ1-5
Donor 2	Positive	TRAV17	CATDPTSLYGNKLVF	TRAJ47	TRBV20-1	CSARTGSSYNSPLHF	TRBJ1-6
Donor 2	Positive	TRAV21	CAVRDYTGTASKLTF	TRAJ44	TRBV23-1	CALPGVAGITYEQYF	TRBJ2-7
Donor 2	Positive	TRAV29	CAASAQGGTSYGKLTF	TRAJ52	TRBV25-1	CASRGWTGPVETQYF	TRBJ2-5
Donor 2	Positive	TRAV22	CAVLLTSGTYKYIF	TRAJ40	TRBV27	CASSLGLAGANEQFF	TRBJ2-1
Donor 2	Positive	TRAV27	CSGGSHSRLMF	TRAJ31	TRBV27	CASSFTAYYGYTF	TRBJ1-2
Donor 2	Positive	TRAV35	CAGASYNNNDMRF	TRAJ43	TRBV27	CASSSAWTGYEQYF	TRBJ2-7
Donor 2	Positive	TRAV8-6	CASHSGGYQKVTF	TRAJ13	TRBV27	CASRGSGNEQFF	TRBJ2-1
Donor 2	Positive	TRAV22	CAVWWTSGSRLTF	TRAJ58	TRBV4-1	CASSQWGLLTDTQYF	TRBJ2-3
Donor 2	Positive	TRAV38-1	CALNSGGSNYKLTF	TRAJ53	TRBV5-4	CASSLAGWQGDDYGYTF	TRBJ1-2
Donor 2	Positive	TRAV19	CALSEALGDKIIF	TRAJ30	TRBV5-8	CASSFVGDTQYF	TRBJ2-3
Donor 2	Positive	TRAV17	CATGGGNTPLVF	TRAJ29	TRBV7-8	CASSPSRSPYEQYF	TRBJ2-7
Donor 2	Positive	TRAV12-2	CAVNSAGKSTF	TRAJ27	TRBV7-9	CASTLYGNEQFF	TRBJ2-1
Donor 2	Positive	TRAV12-2	CAVNSAGKSTF	TRAJ27	TRBV7-9	CASTLYGNEQFF	TRBJ2-1
Donor 2	Positive	TRAV12-2	CAVNSAGKSTF	TRAJ27	TRBV7-9	CASTLYGNEQFF	TRBJ2-1
Donor 2	Positive	TRAV12-2	CAVNSAGKSTF	TRAJ27	TRBV7-9	CASTLYGNEQFF	TRBJ2-1
Donor 2	Positive	TRAV12-2	CAVNSAGKSTF	TRAJ27	TRBV7-9	CASTLYGNEQFF	TRBJ2-1
Donor 2	Positive	TRAV12-2	CAVNSAGKSTF	TRAJ27	TRBV7-9	CASTLYGNEQFF	TRBJ2-1
Donor 2	Positive	TRAV25	CAGGSTLGRLYF	TRAJ18	TRBV7-9	CASSPHGGGTDTQYF	TRBJ2-3
Donor 3	Positive	TRAV21	CAVKGGSEKLVF	TRAJ57	TRBV10-3	CAISPLTSWGTDTQYF	TRBJ2-3
Donor 3	Positive	TRAV27	CTDMPSAGHILTF	TRAJ39	TRBV11-1	CASSRRTGGTEAFF	TRBJ1-1
Donor 3	Positive	TRAV29	CAASATGGSYIPTF	TRAJ6	TRBV11-2	CASSSLAGQIDEQFF	TRBJ2-1
Donor 3	Positive	TRAV19	CALNSNTGTASKLTF	TRAJ44	TRBV19	CASTDLSYGYTF	TRBJ1-2
Donor 3	Positive	TRAV35	CAGLAYTGTASKLTF	TRAJ44	TRBV19	CASSSAGAKNIQYF	TRBJ2-4
Donor 3	Positive	TRAV38-1	CAFMKHVGTASKLTF	TRAJ44	TRBV19	CASRDQRDRVKTEAFF	TRBJ1-1
Donor 3	Positive	TRAV38-1	CAFMKHWGTGNQFYF	TRAJ49	TRBV19	CASSILGDSSGNTIYF	TRBJ1-3
Donor 3	Positive	TRAV38-1	CAFRPEKLVF	TRAJ57	TRBV19	CASSIAGGASYEQYF	TRBJ2-7
Donor 3	Positive	TRAV5	CADRMKGTYKYIF	TRAJ40	TRBV19	CASSARRGNTEAFF	TRBJ1-1
Donor 3	Positive	TRAV8-3	CAVGALTGTASKLTF	TRAJ44	TRBV19	CASSTLTSGTYNEQFF	TRBJ2-1
Donor 3	Positive	TRAV10	CVVSAWDDKIIF	TRAJ30	TRBV2	CASSWGNTGELFF	TRBJ2-2
Donor 3	Positive	TRAV14	CAMRSMIYNQGGKLIF	TRAJ23	TRBV2	CASSGHTQGTYGYTF	TRBJ1-2
Donor 3	Positive	TRAV38-1	CAYSLYTGTASKLTF	TRAJ44	TRBV20-1	CSALGGQASTDTQYF	TRBJ2-3
Donor 3	Positive	TRAV19	CALSVYYGGYQKVPF	TRAJ13	TRBV24-1	CATSGTGYEQYF	TRBJ2-7
Donor 3	Positive	TRAV17	CATDAWSNTGKLIF	TRAJ37	TRBV27	CASSSPPAEQFF	TRBJ2-1
Donor 3	Positive	TRAV21	CAVKEIYNQGGKLIF	TRAJ23	TRBV27	CASSLAYSGANVLTF	TRBJ2-6
Donor 3	Positive	TRAV19	CALSEALNNARLMF	TRAJ31	TRBV28	CASSFRGPLSNEKLFF	TRBJ1-4
Donor 3	Positive	TRAV19	CALSELLTSGTYKYIF	TRAJ40	TRBV28	CASSSLRTPNSPLHF	TRBJ1-6
Donor 3	Positive	TRAV9-2	CALSDQSGGSYIPTF	TRAJ6	TRBV28	CASRRGSGMYEQFF	TRBJ2-1
Donor 3	Positive	TRAV14	CAMREGTGGFKTIF	TRAJ9	TRBV3-1	CASSQSTGVATNEKLFF	TRBJ1-4
Donor 3	Positive	TRAV8-1	CAVYSGGYQKVTF	TRAJ13	TRBV3-1	CASSQVIQTEAFF	TRBJ1-1
Donor 3	Positive	TRAV8-3	CAVGVSGGYNKLIF	TRAJ4	TRBV3-1	CASSQGRGLRNEQFF	TRBJ2-1
Donor 3	Positive	TRAV23	CAAIPSNSGYALNF	TRAJ41	TRBV5-4	CASSPVVGGTGELFF	TRBJ2-2
Donor 3	Positive	TRAV8-2	CVVSGGNSGNTPLVF	TRAJ29	TRBV7-8	CASSLSLTSPRVDEQYF	TRBJ2-7
Donor 3	Positive	TRAV8-2	CVVSGGNSGNTPLVF	TRAJ29	TRBV7-8	CASSLSLTSPRVDEQYF	TRBJ2-7
Donor 3	Positive	TRAV41	CAVRSNTDKLIF	TRAJ34	TRBV7-9	CASSTWGLPRETQYF	TRBJ2-5

Supplementary Table 2

HLA-A2- IMNDMPIYM clones

Donor	HLA-A0201	TRAV	CDR3	TRAJ	TRBV	CDR3	TRBJ
Donor 4	Negative	TRAV12-3	CAMTGGATNKLIF	TRAJ32	TRBV11-2	CASSSSGTGYLDEQFF	TRBJ2-1
Donor 4	Negative	TRAV12-2	CAVRKNNARLMF	TRAJ31	TRBV11-3	CASRGQNTGELFF	TRBJ2-2
Donor 4	Negative	TRAV19	CALGGSWGKLQF	TRAJ24	TRBV11-3	CASSFGMNTEAFF	TRBJ1-1
Donor 4	Negative	TRAV38-1	CAFMIPSYNNNDMRF	TRAJ43	TRBV11-3	CASSLDGTGGSLDEQFF	TRBJ2-1
Donor 4	Negative	TRAV14	CAMRKQPGTSYGKLTF	TRAJ52	TRBV12-5	CAIVFSLPDTQYF	TRBJ2-3
Donor 4	Negative	TRAV12-3	CAMSGGLGNEKLTF	TRAJ48	TRBV19	CASSIWDSQPQHF	TRBJ1-5
Donor 4	Negative	TRAV19	CALSEFTGTASKLTF	TRAJ44	TRBV19	CASSMGAPPTQYF	TRBJ2-3
Donor 4	Negative	TRAV41	CAVNTDKLIF	TRAJ34	TRBV19	CASSWLQGPEAFF	TRBJ1-1
Donor 4	Negative	TRAV8-1	CAVRGGSYIPTF	TRAJ6	TRBV19	CASSRLAGGTDTQYF	TRBJ2-3
Donor 4	Negative	TRAV12-2	CAVWEGNKLVF	TRAJ47	TRBV20-1	CSARDPGGLYYGYTF	TRBJ1-2
Donor 4	Negative	TRAV12-1	CVVMLSNNNARLMF	TRAJ31	TRBV20-1	CSARGEWDRGYF	TRBJ2-7
Donor 4	Negative	TRAV12-1	CVVDDTGFQKLVF	TRAJ8	TRBV20-1	CSAERIGLAGEDYEQYF	TRBJ2-7
Donor 4	Negative	TRAV29	CAASQGGSEKLVF	TRAJ57	TRBV24-1	CATSDSDRGRHGELFF	TRBJ2-2
Donor 4	Negative	TRAV14	CAIRSSGTYKYIF	TRAJ40	TRBV27	CASSLSGGNEKLFF	TRBJ1-4
Donor 4	Negative	TRAV21	CAVPFTDSWGKLQF	TRAJ24	TRBV27	CASNGQGSTEQYF	TRBJ2-7
Donor 4	Negative	TRAV14	CAMRGPLNDYKLSF	TRAJ20	TRBV28	CASSINRGVNTEAFF	TRBJ1-1
Donor 4	Negative	TRAV25	CAHLTGNQFYF	TRAJ49	TRBV28	CASSSPYTQYF	TRBJ2-3
Donor 4	Negative	TRAV38-1	CAFMKLYNSGGSNYKLTF	TRAJ53	TRBV28	CASSSPGTGPSYNEQFF	TRBJ2-1
Donor 4	Negative	TRAV8-2	CAVSGPDGLSF	TRAJ20	TRBV28	CASSSTGGEQYF	TRBJ2-7
Donor 4	Negative	TRAV19	CALRGITQGGSEKLVF	TRAJ57	TRBV3-1	CASSQGQIYGYTF	TRBJ1-2
Donor 4	Negative	TRAV19	CALRGAPYNNNDMRF	TRAJ43	TRBV4-1	CASSHVMGPGQGGTGELFF	TRBJ2-2
Donor 4	Negative	TRAV14	CAMREGPDGQKLLF	TRAJ16	TRBV4-2	CASNQGQAFEQYF	TRBJ2-7
Donor 4	Negative	TRAV8-3	CAVGAYSGAGSYQLTF	TRAJ28	TRBV5-4	CASSLDVYSYEQYF	TRBJ2-7
Donor 4	Negative	TRAV9-2	CALSFPSGGYQKVTF	TRAJ13	TRBV5-6	CASRGRVAMNTEAFF	TRBJ1-1
Donor 4	Negative	TRAV19	CALSEASDRDDKIIF	TRAJ30	TRBV6-5	CASSYGAVAYEQYF	TRBJ2-7
Donor 4	Negative	TRAV41	CAVKADYGGATNKLIF	TRAJ32	TRBV6-5	CASSYGAVAYEQYF	TRBJ2-7
Donor 4	Negative	TRAV4	CLVARPPYNNNDMRF	TRAJ43	TRBV7-2	CASSFAAPGGETQYF	TRBJ2-5
Donor 4	Negative	TRAV12-2	CAANNAGNMLTF	TRAJ39	TRBV7-9	CASNPHQHTEAFF	TRBJ1-1
Donor 4	Negative	TRAV12-2	CAANNAGNMLTF	TRAJ39	TRBV7-9	CASNPHQHTEAFF	TRBJ1-1
Donor 4	Negative	TRAV12-2	CAANNAGNMLTF	TRAJ39	TRBV7-9	CASNPHQHTEAFF	TRBJ1-1
Donor 4	Negative	TRAV12-2	CAANNAGNMLTF	TRAJ39	TRBV7-9	CASNPHQHTEAFF	TRBJ1-1
Donor 4	Negative	TRAV12-2	CAANNAGNMLTF	TRAJ39	TRBV7-9	CASNPHQHTEAFF	TRBJ1-1
Donor 5	Negative	TRAV19	CALNTGTASKLTF	TRAJ44	TRBV11-2	CASSLGVSSYNEQFF	TRBJ2-1
Donor 5	Negative	TRAV10	CVVSPNTGFQKLVF	TRAJ8	TRBV12-3	CASSGGGRDSGYEQYF	TRBJ2-7
Donor 5	Negative	TRAV8-1	CAVNAWSGGYQKVTF	TRAJ13	TRBV12-3	CASRERGDQPQHF	TRBJ1-5
Donor 5	Negative	TRAV1-1	CAVTHYGGATNKLIF	TRAJ32	TRBV12-4	CASSPPGKDTQYF	TRBJ2-3
Donor 5	Negative	TRAV4	CLVGASAAGNKLTF	TRAJ17	TRBV13	CASSLGQGGFGYTF	TRBJ1-2
Donor 5	Negative	TRAV19	CALSDLNAGGTSYGKLTF	TRAJ52	TRBV14	CASSTGALNYGYTF	TRBJ1-2
Donor 5	Negative	TRAV41	CAVLTEKLVF	TRAJ57	TRBV18	CASSPLVYNEQFF	TRBJ2-1
Donor 5	Negative	TRAV25	CAGDRGSTLGRLYF	TRAJ18	TRBV19	CASIRQQGLNEKLFF	TRBJ1-4
Donor 5	Negative	TRAV35	CAGQLYTGTASKLTF	TRAJ44	TRBV19	CASWGAGGKNTGELFF	TRBJ2-2
Donor 5	Negative	TRAV8-1	CAVNTYTGTASKLTF	TRAJ44	TRBV19	CASSAQGGEQFF	TRBJ2-1
Donor 5	Negative	TRAV8-1	CAVNAGSGGGADGLTF	TRAJ45	TRBV19	CASSIDAGLYNEQFF	TRBJ2-1
Donor 5	Negative	TRAV8-1	CAVTAFRGSQGNLIF	TRAJ42	TRBV19	CASRGLLHF	TRBJ1-2
Donor 5	Negative	TRAV25	CAETDSWGKLQF	TRAJ24	TRBV2	CASRLLAAYEQYF	TRBJ2-7
Donor 5	Negative	TRAV26-2	CITITSGSARQLTF	TRAJ22	TRBV2	CASRWTADYGYTF	TRBJ1-2
Donor 5	Negative	TRAV26-2	CILAGGSNYKLTF	TRAJ53	TRBV2	CASSKGQSNYGYTF	TRBJ1-2
Donor 5	Negative	TRAV4	CLVGVPWNSGNTPLVF	TRAJ29	TRBV2	CASSGTENTEAFF	TRBJ1-1
Donor 5	Negative	TRAV5	CAEFLRNNNARLMF	TRAJ31	TRBV2	CASLDWTGSEAFF	TRBJ1-1
Donor 5	Negative	TRAV12-2	CAVNAPPVTF	TRAJ13	TRBV20-1	CSAPSNYGYTF	TRBJ1-2
Donor 5	Negative	TRAV14	CAMRVVIGTSYGKLTF	TRAJ52	TRBV20-1	CSARPRGVEYEQYF	TRBJ2-7
Donor 5	Negative	TRAV19	CALSGVTTDSWGKLQF	TRAJ24	TRBV24-1	CATSDSGQAGDNEQFF	TRBJ2-1
Donor 5	Negative	TRAV12-2	CAVSWDMRF	TRAJ43	TRBV27	CATRGGSSYEQYF	TRBJ2-7
Donor 5	Negative	TRAV3	CAVRGHHADSSYKLIF	TRAJ12	TRBV27	CASSLSSGGEYGYTF	TRBJ1-2
Donor 5	Negative	TRAV29	CAASGVWVRF	TRAJ43	TRBV28	CASSPGTGNRETQYF	TRBJ2-5
Donor 5	Negative	TRAV19	CAQTGANSKLTF	TRAJ56	TRBV3-1	CASSQDPGAYNEQFF	TRBJ2-1
Donor 5	Negative	TRAV19	CALSEALGSQGNLIF	TRAJ42	TRBV4-3	CASSQDGAGYTEAFF	TRBJ1-1
Donor 5	Negative	TRAV1-2	CAVRDPKAGTALIF	TRAJ15	TRBV6-4	CASSDSGSEQFF	TRBJ2-1
Donor 5	Negative	TRAV12-2	CAVREDMRF	TRAJ43	TRBV7-9	CASSLRGSSYEQYF	TRBJ2-7
Donor 6	Negative	TRAV12-2	CAVNMNDMRF	TRAJ43	TRBV10-3	CASQGGFGSKNIQYF	TRBJ2-4
Donor 6	Negative	TRAV8-1	CAVIAPGSARQLTF	TRAJ22	TRBV10-3	CAIRRDSGELFF	TRBJ2-2
Donor 6	Negative	TRAV12-2	CAVPTYKYIF	TRAJ40	TRBV11-2	CASGWSHEQYF	TRBJ2-7
Donor 6	Negative	TRAV8-6	CAVSEHQLTF	TRAJ53	TRBV11-2	CASSSYRDGNNEQFF	TRBJ2-1
Donor 6	Negative	TRAV19	CALSEALASGTYKYIF	TRAJ40	TRBV12-3	CASSDRDRVFFGANVLTF	TRBJ2-6
Donor 6	Negative	TRAV19	CALSVFTGTASKLTF	TRAJ44	TRBV19	CASSPTPGESSGGYTF	TRBJ1-2
Donor 6	Negative	TRAV19	CALSVFRGTASKLTF	TRAJ44	TRBV19	CASQGVDEQFF	TRBJ2-1
Donor 6	Negative	TRAV19	CAMSEARGAGNKLTF	TRAJ17	TRBV19	CASSSRTSGTTDTQYF	TRBJ2-3
Donor 6	Negative	TRAV38-1	CAFMKHTGTASKLTF	TRAJ44	TRBV19	CASSPQGARHGYTF	TRBJ1-2
Donor 6	Negative	TRAV16	CALRVKWGGFKTIF	TRAJ9	TRBV2	CASSDPRGGTDTQYF	TRBJ2-3
Donor 6	Negative	TRAV17	CISTDSWGKLQF	TRAJ24	TRBV2	CASLGRQNTEAFF	TRBJ1-1
Donor 6	Negative	TRAV17	CDKYNNNDMRF	TRAJ43	TRBV2	CASSGPTSGGAYNEQFF	TRBJ2-1
Donor 6	Negative	TRAV8-6	CAVRNYGQNFVF	TRAJ26	TRBV2	CAIIFPGSSYEQYF	TRBJ2-7
Donor 6	Negative	TRAV19	CALSGPGRDDKIIF	TRAJ30	TRBV24-1	CATSGPGQGPYNEQFF	TRBJ2-1
Donor 6	Negative	TRAV12-2	CAVNQFYF	TRAJ49	TRBV25-1	CASSGMGPPLHF	TRBJ1-6
Donor 6	Negative	TRAV8-6	CAVSAGPAGGTSYGKLTF	TRAJ52	TRBV25-1	CASSEYQRGEAFF	TRBJ1-1
Donor 6	Negative	TRAV12-2	CAVSARLMF	TRAJ31	TRBV27	CASSPRQGGHEQYF	TRBJ2-7
Donor 6	Negative	TRAV12-3	CAMSGVGGTYKYIF	TRAJ40	TRBV27	CASRGIMNTGELFF	TRBJ2-2
Donor 6	Negative	TRAV26-1	CIVRVPWDKLIF	TRAJ32	TRBV27	CASSPAGPGDEQYF	TRBJ2-7
Donor 6	Negative	TRAV12-2	CAVLDGQKLLF	TRAJ16	TRBV28	CASSPGGRQYF	TRBJ2-7
Donor 6	Negative	TRAV20	CAVRVYSGGYQKVTF	TRAJ13	TRBV4-1	CAVGSGATDTQYF	TRBJ2-3
Donor 6	Negative	TRAV19	CALKAAAGNKLTF	TRAJ17	TRBV7-2	CASSIRQFTDTQYF	TRBJ2-3
Donor 6	Negative	TRAV4	CLVGHRGASNSGYALNF	TRAJ41	TRBV7-2	CASGHPGGYNEQFF	TRBJ2-1
Donor 6	Negative	TRAV12-2	CAVTGKLIF	TRAJ23	TRBV7-9	CASSPNTEAFF	TRBJ1-1
Donor 6	Negative	TRAV12-2	CAGNDMRF	TRAJ43	TRBV7-9	CASSGWETGYGYTF	TRBJ1-2
Donor 6	Negative	TRAV8-2	CAVSVNTGTASKLTF	TRAJ44	TRBV7-9	CASSGRGEKLFF	TRBJ1-4
Donor 6	Negative	TRAV35	CAGISGNQFYF	TRAJ49	TRBV9	CASSVTGLPGEPQHF	TRBJ1-5

Supplementary Table 3

HLA-0201+ FLCMKALLL clones

Donor	HLA-A0201	TRAV	CDR3	TRAJ	TRBV	CDR3	TRBJ
Donor 7	Positive	TRAV1-2	CAVRDSDYKLSF	TRAJ20	TRBV6-4	CASSESGDGTDTQYF	TRBJ2-3
Donor 7	Positive	TRAV12-1	CVVNIYDFGNEKLTF	TRAJ48	TRBV20-1	CSAKSLVGGYGYTF	TRBJ1-2
Donor 7	Positive	TRAV12-2	CAVTFYGNNRLAF	TRAJ7	TRBV7-8	CASSPKSGSGELFF	TRBJ2-2
Donor 7	Positive	TRAV12-2	CAVTFYGNNRLAF	TRAJ7	TRBV7-2	CASSPKSGSGELFF	TRBJ2-2
Donor 7	Positive	TRAV12-2	CAVLYSGAGSYQLTF	TRAJ28	TRBV19	CASSITPAGGTEAFF	TRBJ1-1
Donor 7	Positive	TRAV12-2	CAVLDSSYKLIF	TRAJ12	TRBV4-1	CASSQDDSARTQFF	TRBJ2-1
Donor 7	Positive	TRAV14	CAMREGSYSNFGNEKLTF	TRAJ48	TRBV20-1	CRAGGGGLRPTQYF	TRBJ2-3
Donor 7	Positive	TRAV17	CATHMNYGGSQGNLIF	TRAJ42	TRBV4-2	CASSQDEGAGTEAFF	TRBJ1-1
Donor 7	Positive	TRAV19	CALSEATGGFKTIF	TRAJ9	TRBV20-1	CSAEGLSPEAFF	TRBJ1-1
Donor 7	Positive	TRAV21	CAVKVLYGNKLVF	TRAJ47	TRBV7-9	CASSLKMPSGVSRTDTQYF	TRBJ2-3
Donor 7	Positive	TRAV21	CAVPFLTGFQKLVF	TRAJ8	TRBV27	CASSLSVDQETQYF	TRBJ2-5
Donor 7	Positive	TRAV2	CAASIGNFGNEKLTF	TRAJ48	TRBV4-2	CASSPQRNTEAFF	TRBJ1-1
Donor 7	Positive	TRAV23	CAASIGNFGNEKLTF	TRAJ48	TRBV4-2	CASSPSRNTEAFF	TRBJ1-1
Donor 7	Positive	TRAV27	CAGEGANNLFF	TRAJ36	TRBV27	CASSPSVDSLNQPQHF	TRBJ1-5
Donor 7	Positive	TRAV27	CAGENEKLTF	TRAJ48	TRBV25-1	CASSVTGTEAFF	TRBJ1-1
Donor 7	Positive	TRAV29	CAASAQAQKLVF	TRAJ54	TRBV4-2	CASSQSGGSTDTQYF	TRBJ2-3
Donor 7	Positive	TRAV34	CGADTGNQFYF	TRAJ49	TRBV23-1	CASTGQSYEQYF	TRBJ2-7
Donor 7	Positive	TRAV34	CGADTGNQFYF	TRAJ49	TRBV5-1	CASSVPVAGNTLTDTQYF	TRBJ2-3
Donor 7	Positive	TRAV8-2	CVVSDLSGNQFYF	TRAJ49	TRBV27	CASSLSSARNYNEQFF	TRBJ2-1
Donor 7	Positive	TRAV8-2	CAVSDPLNFGNEKLTF	TRAJ48	TRBV13	CASSDPFSGNTIYF	TRBJ1-3
Donor 7	Positive	TRAV8-6	CAVSDQYNNARLMF	TRAJ31	TRBV27	CASSFEQGDGDTQYF	TRBJ2-3
Donor 8	Positive	TRAV22	CAVAPDYKLSF	TRAJ20	TRBV4-1	CASSQDLGETQYF	TRBJ2-5
Donor 8	Positive	TRAV24	CAFHDYKLSF	TRAJ20	TRBV29-1	CSVEDEGPDGYTF	TRBJ1-2
Donor 8	Positive	TRAV24	CAFSDSWGKFQF	TRAJ24	TRBV4-2	CASSQDVDRSSPLHF	TRBJ1-6
Donor 8	Positive	TRAV29	CAASAILNARLMF	TRAJ31	TRBV27	CASSPDSLNEKLFF	TRBJ1-4
Donor 8	Positive	TRAV29	CAASEPPDGGTSYGKLTF	TRAJ52	TRBV7-2	CASSLGAEASQETQYF	TRBJ2-5
Donor 8	Positive	TRAV38	CSLTQGGSEKLVF	TRAJ57	TRBV25-1	CASSSGETQYF	TRBJ2-5
Donor 8	Positive	TRAV4	CLVGDQGFGNEKLTF	TRAJ48	TRBV3-1	CASSQAAGIYNEQFF	TRBJ2-1
Donor 8	Positive	TRAV5	CAESRGKLVF	TRAJ47	TRBV20-1	CSASWAGSVEGETQYF	TRBJ2-5
Donor 8	Positive	TRAV5	CAESRGKLVF	TRAJ47	TRBV20-1	CSASWAGSVEGETQYF	TRBJ2-5
Donor 8	Positive	TRAV5	CAESRGKLVF	TRAJ47	TRBV20-1	CSASWAGSVEGETQYF	TRBJ2-5
Donor 8	Positive	TRAV5	CAESRGKLVF	TRAJ47	TRBV20-1	CSASWAGSVEGETQYF	TRBJ2-5
Donor 8	Positive	TRAV5	CAESRGKLVF	TRAJ47	TRBV20-1	CSASWAGSVEGETQYF	TRBJ2-5
Donor 8	Positive	TRAV5	CAESRGKLVF	TRAJ47	TRBV20-1	CSASWAGSVEGETQYF	TRBJ2-5
Donor 8	Positive	TRAV5	CAESRGKLVF	TRAJ47	TRBV20-1	CSASWAGSVEGETQYF	TRBJ2-5
Donor 8	Positive	TRAV5	CAESRGKLVF	TRAJ47	TRBV20-1	CSASWAGSVEGETQYF	TRBJ2-5
Donor 8	Positive	TRAV5	CAESRGKLVF	TRAJ47	TRBV20-1	CSASWAGSVEGETQYF	TRBJ2-5
Donor 8	Positive	TRAV5	CAESRGKLVF	TRAJ47	TRBV20-1	CSASWAGSVEGETQYF	TRBJ2-5
Donor 8	Positive	TRAV5	CAESRGKLVF	TRAJ47	TRBV20-1	CSASWAGSVEGETQYF	TRBJ2-5
Donor 8	Positive	TRAV5	CAESRGKLVF	TRAJ47	TRBV20-1	CSASWAGSVEGETQYF	TRBJ2-5
Donor 8	Positive	TRAV5	CAESRGKLVF	TRAJ47	TRBV20-1	CSASWAGSVEGETQYF	TRBJ2-5
Donor 8	Positive	TRAV5	CAESRGKLVF	TRAJ47	TRBV20-1	CSASWAGSVEGETQYF	TRBJ2-5
Donor 8	Positive	TRAV5	CAESRGKLVF	TRAJ47	TRBV20-1	CSASWAGSVEGETQYF	TRBJ2-5
Donor 8	Positive	TRAV5	CAESRGKLVF	TRAJ47	TRBV20-1	CSASWAGSVEGETQYF	TRBJ2-5
Donor 8	Positive	TRAV5	CAESRGKLVF	TRAJ47	TRBV20-1	CSASWAGSVEGETQYF	TRBJ2-5
Donor 8	Positive	TRAV5	CAESRGKLVF	TRAJ47	TRBV20-1	CSASWAGSVEGETQYF	TRBJ2-5
Donor 8	Positive	TRAV5	CAESRGKLVF	TRAJ47	TRBV20-1	CSASWAGSVEGETQYF	TRBJ2-5
Donor 8	Positive	TRAV5	CAESRGKLVF	TRAJ47	TRBV20-1	CSASWAGSVEGETQYF	TRBJ2-5
Donor 8	Positive	TRAV5	CAESRGKLVF	TRAJ47	TRBV20-1	CSASWAGSVEGETQYF	TRBJ2-5
Donor 8	Positive	TRAV5	CAESRGKLVF	TRAJ47	TRBV20-1	CSASWAGSVEGETQYF	TRBJ2-5
Donor 8	Positive	TRAV5	CAESRGKLVF	TRAJ47	TRBV20-1	CSASWAGSVEGETQYF	TRBJ2-5
Donor 8	Positive	TRAV8-1	CVVSRGAGNMLTF	TRAJ39	TRBV2	CASSDLNNSPLHF	TRBJ1-6
Donor 8	Positive	TRAV8-2	CVVSGSGYALNF	TRAJ41	TRBV6-4	CASRGQGYSNQPQHF	TRBJ1-5
Donor 8	Positive	TRAV8-6	CAVSVKDSGYSTLTF	TRAJ11	TRBV4-1	CASSQHGGATDTQYF	TRBJ2-3
Donor 9	Positive	TRAV9-2	CALSDPGGGAGSYQLTF	TRAJ28	TRBV4-2	CASSQDQGDYYGYTF	TRBJ1-2
Donor 9	Positive	TRAV1-2	CGCMVDSWGKLQF	TRAJ24	TRBV28	CASSPFGQGAGELFF	TRBJ2-2
Donor 9	Positive	TRAV1-2	CGCMVDSWGKLQF	TRAJ24	TRBV28	CASSPFRQGAGELFF	TRBJ2-2
Donor 9	Positive	TRAV12-1	CVVNDVDSSYKLIF	TRAJ12	TRBV13	CASSLPSYNEQFF	TRBJ2-1
Donor 9	Positive	TRAV12-2	CAVIGNTGNQFYF	TRAJ49	TRBV9	CASGTGGNEETQYF	TRBJ2-5
Donor 9	Positive	TRAV14	CAMSIISNFGNEKLTF	TRAJ48	TRBV27	CASSLYSGTGVNQPQHF	TRBJ1-5
Donor 9	Positive	TRAV14	CAMRDYNQGGKLIF	TRAJ23	TRBV27	CASSLLGVDSFYNNEQFF	TRBJ2-1
Donor 9	Positive	TRAV14	CAESSSNTGKLIF	TRAJ37	TRBV29-1	CSVPVQGEQFF	TRBJ2-1
Donor 9	Positive	TRAV16	CDNFNKFYF	TRAJ21	TRBV2	CASEDGNYNEQFF	TRBJ2-1
Donor 9	Positive	TRAV19	CALSEAYAGGTSYGKLTF	TRAJ52	TRBV20-1	CSARGSKGGGQKNEQF	TRBJ2-1
Donor 9	Positive	TRAV19	CALNTNTGFQKLVF	TRAJ8	TRBV27	CASSIGQQGAYNEQFF	TRBJ2-1
Donor 9	Positive	TRAV20	CAVRTEFSGTYKYIF	TRAJ40	TRBV12-4	CASSVTGTGPSNEKLFF	TRBJ1-4
Donor 9	Positive	TRAV21	CAVENGGFKTIF	TRAJ9	TRBV11-3	CASSRTGEWDEQFF	TRBJ2-1
Donor 9	Positive	TRAV21	CAVDPGGGADGLTF	TRAJ45	TRBV12-4	CASGGTGLNYGYTF	TRBJ1-2
Donor 9	Positive	TRAV27	CAGEGTTDSWGKLQF	TRAJ24	TRBV2	CASKYVPDPYSPLHF	TRBJ1-6
Donor 9	Positive	TRAV27	CAGVPYFGNEKLTF	TRAJ48	TRBV27	CASSSAVNYGYTF	TRBJ1-2
Donor 9	Positive	TRAV29	CAASEANDMRF	TRAJ43	TRBV10-3	CAISESYTEAFF	TRBJ1-1
Donor 9	Positive	TRAV3	CAVRPRDTDKLIF	TRAJ34	TRBV19	CASSIGGRTEAFF	TRBJ1-1
Donor 9	Positive	TRAV35	CAGQPLGGAQKLVF	TRAJ54	TRBV29-1	CSVEGDNPGYTF	TRBJ1-2
Donor 9	Positive	TRAV36	CAPSGGGADGLTF	TRAJ45	TRBV29-1	CSVEDLGAGYTF	TRBJ1-2
Donor 9	Positive	TRAV4	CLVGDPGGRYGGSQGNLIF	TRAJ42	TRBV30	CAWSVDSDNTGELFF	TRBJ2-2
Donor 9	Positive	TRAV4	CLVGDPGGRYGGSQGNLIF	TRAJ42	TRBV30	CAWSVDSDNTGELFF	TRBJ2-2
Donor 9	Positive	TRAV4	CLVGDPGGRYGGSQGNLIF	TRAJ42	TRBV30	CAWSVDSDNTGELFF	TRBJ2-2
Donor 9	Positive	TRAV4	CLVGDPGGRYGGSQGNLIF	TRAJ42	TRBV30	CAWSVDSDNTGELFF	TRBJ2-2
Donor 9	Positive	TRAV41	CAVRENYQLIW	TRAJ33	TRBV19	CASSLTGGEQYF	TRBJ2-7
Donor 9	Positive	TRAV41	CAPQGDQGNLIF	TRAJ42	TRBV28	CASISGIFDNSPLHF	TRBJ1-6
Donor 9	Positive	TRAV41	CAVRENYQLIW	TRAJ33	TRBV7-3	CASSLGATFTDTQYF	TRBJ2-3
Donor 9	Positive	TRAV5	CAVDSSYKLIF	TRAJ12	TRBV12-4	CASRDDNQETQYF	TRBJ2-5
Donor 9	Positive	TRAV5	CAETYPYGGSQGNLIF	TRAJ42	TRBV2	CASLGEATEAFF	TRBJ1-1
Donor 9	Positive	TRAV8-1	CAVKQLNNAGNMLTF	TRAJ39	TRBV29-1	CSVEGLSEQFF	TRBJ2-1
Donor 9	Positive	TRAV8-2	CVVLTGTASKLTF	TRAJ44	TRBV7-9	CASSLEIDGAETQYF	TRBJ2-5
Donor 9	Positive	TRAV9-2	CVPWGRPGMRF	TRAJ43	TRBV19	CASSLGGENSPLHF	TRBJ1-6

Supplementary Table 4

HLA-0201+ FLCMKALLL clones

Donor	HLA-A0201	TRAV	CDR3	TRAJ	TRBV	CDR3	TRBJ
Donor 10	Negative	TRAV1-1	CAVSNYQLIW	TRAJ33	TRBV27	CASSLGTGQGNEQFF	TRBJ2-1
Donor 10	Negative	TRAV1-2	CAVNGNARLMF	TRAJ31	TRBV29-1	CSVEDGRTDTQYF	TRBJ2-3
Donor 10	Negative	TRAV1-2	CAVRGSWGKFQF	TRAJ24	TRBV10-1	CASSEPSGPDNEQFF	TRBJ2-1
Donor 10	Negative	TRAV12-2	CAVASDGNNRKLIW	TRAJ38	TRBV9	CASSVWAEDQPQHF	TRBJ1-5
Donor 10	Negative	TRAV12-3	CAWGGSNYQLIW	TRAJ33	TRBV3-1	CASSPGASLGNEKLFF	TRBJ1-4
Donor 10	Negative	TRAV12-3	CAMSMYSGGGADGLTF	TRAJ45	TRBV4-2	CASSPSGGASADTQYF	TRBJ2-3
Donor 10	Negative	TRAV14/DV4	CAMSGGSNYKLTF	TRAJ53	TRBV29-1	CSVEGDRVEQFF	TRBJ2-1
Donor 10	Negative	TRAV21	CAAEGTYKYIF	TRAJ40	TRBV27	CASSTGTGNTEAFF	TRBJ1-1
Donor 10	Negative	TRAV21	CAAEGTYKYIF	TRAJ40	TRBV27	CASSTGTGNTEAFF	TRBJ1-1
Donor 10	Negative	TRAV24	CADGPDYKLSF	TRAJ20	TRBV25-1	CASSEGAGNTIYF	TRBJ1-3
Donor 10	Negative	TRAV26-1	CIVSGDNYGQNFVF	TRAJ26	TRBV13	CASSSLLGYNEQFF	TRBJ2-1
Donor 10	Negative	TRAV35	CAGPSHNTDKLIF	TRAJ34	TRBV27	CASSLIGTGGEKLFF	TRBJ1-4
Donor 10	Negative	TRAV4	CLVGDGYNFNKFYF	TRAJ21	TRBV4-2	CASSQLAAGTDSYEQYF	TRBJ2-7
Donor 10	Negative	TRAV5	CADLTGGGNKLTF	TRAJ10	TRBV25-1	CASGGLGEETQYF	TRBJ2-5
Donor 10	Negative	TRAV8-1	CAVINQGGKLIF	TRAJ23	TRBV23-1	CASSHQDSSSYEQYV	TRBJ2-7
Donor 10	Negative	TRAV8-3	CAVGSLTTDSWGKLQF	TRAJ24	TRBV20-1	CSALEGGYEQYF	TRBJ2-7
Donor 10	Negative	TRAV8-6	CAVSDREGGSEKLVF	TRAJ57	TRBV29-1	CSVVGVWAEAFF	TRBJ1-1
Donor 11	Negative	TRAV1-1	CAVRDLEGGGNKLTF	TRAJ10	TRBV19	CASRKSPQATNEKLFF	TRBJ1-4
Donor 11	Negative	TRAV12-3	CAMSGDRFNKFYF	TRAJ21	TRBV11-3	CASSLVSQGDEQFF	TRBJ2-1
Donor 11	Negative	TRAV13-2	CAEFGGSQGNLIF	TRAJ42	TRBV11-2	CASSPGTYLTDTQYF	TRBJ2-3
Donor 11	Negative	TRAV14/DV4	CAMREASNSGGYQKVTF	TRAJ13	TRBV13	CASSLPLALDTTGEGGYTF	TRBJ1-2
Donor 11	Negative	TRAV14/DV4	CAMRVPNPMIYNQGGKLIF	TRAJ23	TRBV27	CASSLTAGLHSNQPQHF	TRBJ1-5
Donor 11	Negative	TRAV14/DV4	CAMRSLKNSNSGYALNF	TRAJ41	TRBV7-2	CASNNGWNQETQYF	TRBJ2-5
Donor 11	Negative	TRAV17	CATDTGFKTIF	TRAJ9	TRBV11-2	CASSLGSEGYGVETQYF	TRBJ2-5
Donor 11	Negative	TRAV19	CALSEASGNQFYF	TRAJ49	TRBV19	CASALSYNEQFF	TRBJ2-1
Donor 11	Negative	TRAV19	CALSEASGNQFYF	TRAJ49	TRBV19	CASSPNYGEQFF	TRBJ2-1
Donor 11	Negative	TRAV19	CALSEASGNQFYF	TRAJ49	TRBV19	CASSPNYGEQFF	TRBJ2-1
Donor 11	Negative	TRAV19	CALSEASGNQFYF	TRAJ49	TRBV19	CASSPNYGEQFF	TRBJ2-1
Donor 11	Negative	TRAV19	CALSEASGNQFYF	TRAJ49	TRBV19	CASSPNYGEQFF	TRBJ2-1
Donor 11	Negative	TRAV19	CALSEASGNQFYF	TRAJ49	TRBV19	CASSPNYGEQFF	TRBJ2-1
Donor 11	Negative	TRAV19	CALSEASGNQFYF	TRAJ49	TRBV19	CASSPNYGEQFF	TRBJ2-1
Donor 11	Negative	TRAV19	CALSEASGNQFYF	TRAJ49	TRBV19	CASALSYNEQFF	TRBJ2-1
Donor 11	Negative	TRAV19	CALSEASGNQFYF	TRAJ49	TRBV19	CASALSYNEQFF	TRBJ2-1
Donor 11	Negative	TRAV19	CALSEASGNQFYF	TRAJ49	TRBV19	CASSPNYGEQFF	TRBJ2-1
Donor 11	Negative	TRAV19	CALSEASGNQFYF	TRAJ49	TRBV19	CASSPNYGEQFF	TRBJ2-1
Donor 11	Negative	TRAV19	CALSEASGNQFYF	TRAJ49	TRBV19	CASSPNYGEQFF	TRBJ2-1
Donor 11	Negative	TRAV19	CALSEASGNQFYF	TRAJ49	TRBV19	CASSPNYGEQFF	TRBJ2-1
Donor 11	Negative	TRAV19	CALKADTGFQKLVF	TRAJ8	TRBV27	CASSPYPGANVLTF	TRBJ2-6
Donor 11	Negative	TRAV19	CALSGISSGSARQLTF	TRAJ22	TRBV12-3	CASSLGVYEQYF	TRBJ2-7
Donor 11	Negative	TRAV21	CAANNYNTDKLIF	TRAJ34	TRBV19	CASSIYGAGYGTDTQYF	TRBJ2-3
Donor 11	Negative	TRAV21	CAVYTDKLIF	TRAJ34	TRBV28	CASSLIWGTGFSSSYNEQFF	TRBJ2-1
Donor 11	Negative	TRAV27	CAGGFATNAGKSTF	TRAJ27	TRBV4-2	CASSQDPGGLAGAEQFF	TRBJ2-1
Donor 11	Negative	TRAV29/DV5	CAASEVQGAQKLVF	TRAJ54	TRBV10-3	CAIMTSGRARVNTDTQYF	TRBJ2-3
Donor 11	Negative	TRAV3	CAVSIYNTDKLIF	TRAJ34	TRBV27	CASSLVTGTGADTQYF	TRBJ2-3
Donor 11	Negative	TRAV38-1	CAFMAGNTPLVF	TRAJ29	TRBV23-1	CASSSTAANTEAFF	TRBJ1-1
Donor 11	Negative	TRAV8-2	CVVSSNDMRF	TRAJ43	TRBV5-8	CASSFLDLSAGDTQYF	TRBJ2-3
Donor 11	Negative	TRAV8-2	CVVTLNDMRF	TRAJ43	TRBV19	CASDSTDNQPQHF	TRBJ1-5
Donor 11	Negative	TRAV9-2	CALIENDMRF	TRAJ43	TRBV25-1	CASSEFETQYF	TRBJ2-5
Donor 12	Negative	TRAV12-1	CAVDNTDKLIF	TRAJ34	TRBV6-5	CAAGSYEQYF	TRBJ2-7
Donor 12	Negative	TRAV12-1	CAPDNYGQNFVF	TRAJ26	TRBV6-5	CASSLSGGNEAFF	TRBJ1-1
Donor 12	Negative	TRAV12-1	CAVDNTDKLIF	TRAJ34	TRBV11-2	CASSQGGWRDKQYF	TRBJ2-3
Donor 12	Negative	TRAV12-2	CAGYYGQKFVF	TRAJ26	TRBV10-3	CAISEENIDTQYF	TRBJ2-3
Donor 12	Negative	TRAV12-2	CAVNGKDTGRRALTF	TRAJ5	TRBV28	CASSPFGLGDSPLHF	TRBJ1-6
Donor 12	Negative	TRAV12-2	CAVTGFQKLVF	TRAJ8	TRBV4-2	CASSPPSQETQYF	TRBJ2-5
Donor 12	Negative	TRAV12-2	CAFPGGSQGNLIF	TRAJ42	TRBV25-1	CASSWALGEQYF	TRBJ2-7
Donor 12	Negative	TRAV12-2	CAVMLSYGQNFVF	TRAJ26	TRBV29-1	CSAEGNTEAFF	TRBJ1-1
Donor 12	Negative	TRAV12-3	CAGDSGGGGDGLTF	TRAJ45	TRBV29-1	CSVDAGWGGYTF	TRBJ1-2
Donor 12	Negative	TRAV13-1	CAAILYSGGGADGLTF	TRAJ45	TRBV6-5	CASSTGTYNEQFF	TRBJ2-1
Donor 12	Negative	TRAV13-1	CAAIIGGGADGLTF	TRAJ45	TRBV6-5	CASSYGSYNEQFF	TRBJ2-1
Donor 12	Negative	TRAV14	CAMSPRGTGNQFYF	TRAJ49	TRBV29-1	CSVEANRAEQFF	TRBJ2-1
Donor 12	Negative	TRAV16	CALPSTGFQKLVF	TRAJ8	TRBV28	CASSLNEAALGPSEQYF	TRBJ2-7
Donor 12	Negative	TRAV17	CATDLNQAGTALIF	TRAJ15	TRBV19	CASSMSGTGTEAFF	TRBJ1-1
Donor 12	Negative	TRAV17	CATVPNQAGTALIF	TRAJ15	TRBV13	CASSFTVGTEAFF	TRBJ1-1
Donor 12	Negative	TRAV17	CATSLLGFGNVLHC	TRAJ35	TRBV4-1	CASSLGVLGFYEQYF	TRBJ2-7
Donor 12	Negative	TRAV17	CATVVNQAGTALIF	TRAJ15	TRBV19	CASNRGTGNEQFF	TRBJ2-1
Donor 12	Negative	TRAV19	CALSEGYGGSQGNLIF	TRAJ42	TRBV28	CASSSGLYSVF	TRBJ2-1
Donor 12	Negative	TRAV19	CALSEPFPPNFGNEKLTF	TRAJ48	TRBV4-2	CASSLDSGHGMAFF	TRBJ1-1
Donor 12	Negative	TRAV21	CAVLPILTGGGNKLTF	TRAJ10	TRBV11-2	CASSLGGTWGDTQYF	TRBJ2-3
Donor 12	Negative	TRAV22	CAVGLNARLMF	TRAJ31	TRBV13	CASSSYDPGGTGELFF	TRBJ2-2
Donor 12	Negative	TRAV22	CAVAPGYALNF	TRAJ41	TRBV5-4	CASSRRPGQGTYGYTF	TRBJ1-2
Donor 12	Negative	TRAV23	CAAPVNFGNEKLTF	TRAJ48	TRBV29-1	CSVEGNSYEQYF	TRBJ2-7
Donor 12	Negative	TRAV26-1	CIVSLTQGGSEKLVF	TRAJ57	TRBV29-1	CSVDFWVREENYGYTF	TRBJ1-2
Donor 12	Negative	TRAV27	CAGGTSNYGGTHGNLIF	TRAJ42	TRBV2	CASSEWSGVGYEQYF	TRBJ2-7
Donor 12	Negative	TRAV29	CAADSGGGADGLTF	TRAJ45	TRBV13	CASSYGTSGSSGEQYF	TRBJ2-7
Donor 12	Negative	TRAV38-2	CAYGDYKLSF	TRAJ20	TRBV2	CASSPDSYEQYF	TRBJ2-7
Donor 12	Negative	TRAV38-2	CAYRDINTNAGKSTF	TRAJ27	TRBV23-1	CAPERGAEETQYF	TRBJ2-5
Donor 12	Negative	TRAV5	CAEIRSNTGKLIF	TRAJ37	TRBV29-1	CSVVGLNTEAFF	TRBJ1-1
Donor 12	Negative	TRAV5	CAEMSDTGFQKLVF	TRAJ8	TRBV4-1	CASSQGGGVDEQFF	TRBJ2-1
Donor 12	Negative	TRAV6	CALCPRARLMF	TRAJ31	TRBV12-3	CASSLGGPNYGYTF	TRBJ1-2
Donor 12	Negative	TRAV8-2	CVVSDLGATNKLIF	TRAJ32	TRBV27	CASAEGRLSSYEQYF	TRBJ2-7
Donor 12	Negative	TRAV8-2	CVVAATYQLIW	TRAJ33	TRBV6-5	CASSYVPPGGLDYEQYF	TRBJ2-7
Donor 13	Negative	TRAV10	CVVSSIYNTDKLIF	TRAJ34	TRBV12-4	CASSRTEREPQPQHF	TRBJ1-5
Donor 13	Negative	TRAV1-2	CASMDSNYQLIW	TRAJ33	TRBV20-1	CSGKLAGDRNEQFF	TRBJ2-1
Donor 13	Negative	TRAV16	CALDGPSNTGKLIF	TRAJ37	TRBV15	CATSESSGQTYEQYF	TRBJ2-7
Donor 13	Negative	TRAV16	CALDGPSNTGKLIF	TRAJ37	TRBV15	CATSESSGQTYEQYF	TRBJ2-7
Donor 13	Negative	TRAV16	CGGNKLVF	TRAJ47	TRBV3-1	CASSQDFSPYGYTF	TRBJ1-2
Donor 13	Negative	TRAV16	CALDGPSNTGKLIF	TRAJ37	TRBV15	CATSESSGQTYEQYF	TRBJ2-7
Donor 13	Negative	TRAV16	CALDGPSNTGKLIF	TRAJ37	TRBV15	CATSESSGQTYEQYF	TRBJ2-7
Donor 13	Negative	TRAV16	CALDGPSNTGKLIF	TRAJ37	TRBV15	CATSESSGQTYEQYF	TRBJ2-7
Donor 13	Negative	TRAV17	CATDAEGNNRLAF	TRAJ7	TRBV19	CASSIFGGGLGEQFF	TRBJ2-1
Donor 13	Negative	TRAV17	CATDGTSYGKLTF	TRAJ52	TRBV19	CASSRGLAGGNEQFF	TRBJ2-1
Donor 13	Negative	TRAV34	CGAVGYQKVTF	TRAJ13	TRBV2	CALNGEISYNEQFF	TRBJ2-1
Donor 13	Negative	TRAV6	CALPNTGGFKTIF	TRAJ9	TRBV25-1	CASSDTSASNYEQYF	TRBJ2-7
Donor 13	Negative	TRAV8-1	CAVIWYNNNDMRF	TRAJ43	TRBV4-2	CASSQGVNTGELFF	TRBJ2-2
Donor 13	Negative	TRAV8-6	CAVSGLTPSGYSTLTF	TRAJ11	TRBV3-1	CASSQDWGGDNEQFF	TRBJ2-1
Donor 13	Negative	TRAV9-2	CAPPRGSNYKLTF	TRAJ53	TRBV20-1	CSAETGLSYEQYF	TRBJ2-7
